# Bridging the Translational Gap in Heart Failure Research: Using Human iPSC-derived Cardiomyocytes to Accelerate Therapeutic Insights

**DOI:** 10.14797/mdcvj.1295

**Published:** 2023-11-16

**Authors:** Leslye Venegas-Zamora, Matthew Fiedler, William Perez, Francisco Altamirano

**Affiliations:** 1 Houston Methodist Research Institute, Houston, Texas, US; 2 Facultad de Ciencias Químicas y Farmacéuticas, Universidad de Chile, Santiago, Chile; 3 Weill Cornell Graduate School of Medical Sciences, New York, New York, US; 4 Weill Cornell Medical College, New York, New York, US

**Keywords:** heart failure, cardiomyocytes, hypoxia, ischemia and reperfusion injury, stem cells

## Abstract

Heart failure (HF) remains a leading cause of death worldwide, with increasing prevalence and burden. Despite extensive research, a cure for HF remains elusive. Traditionally, the study of HF’s pathogenesis and therapies has relied heavily on animal experimentation. However, these models have limitations in recapitulating the full spectrum of human HF, resulting in challenges for clinical translation. To address this translational gap, research employing human cells, especially cardiomyocytes derived from human-induced pluripotent stem cells (hiPSC-CMs), offers a promising solution. These cells facilitate the study of human genetic and molecular mechanisms driving cardiomyocyte dysfunction and pave the way for research tailored to individual patients. Further, engineered heart tissues combine hiPSC-CMs, other cell types, and scaffold-based approaches to improve cardiomyocyte maturation. Their tridimensional architecture, complemented with mechanical, chemical, and electrical cues, offers a more physiologically relevant environment. This review explores the advantages and limitations of conventional and innovative methods used to study HF pathogenesis, with a primary focus on ischemic HF due to its relative ease of modeling and clinical relevance. We emphasize the importance of a collaborative approach that integrates insights obtained in animal and hiPSC-CMs-based models, along with rigorous clinical research, to dissect the mechanistic underpinnings of human HF. Such an approach could improve our understanding of this disease and lead to more effective treatments.

## Introduction

Heart failure (HF) is a syndrome caused by functional impairment of ventricular blood filling or ejection from the heart, leading to reduced cardiac output. Worldwide cases of HF continue to rise and are expected to reach 8 million in the United States (US) alone by 2030.^1^ Multiple factors contribute to increasing HF prevalence, such as an aging population and improved survival after myocardial infarction (MI). Moreover, globally there is an increasing prevalence of comorbidities such as diabetes, obesity, hypertension, and chronic kidney disease, which are common risk factors for developing HF. Understanding the diverse molecular landscape of HF is crucial, especially given the differing underlying mechanisms and clinical presentations.

The many types of HF are classified based on severity, progression, and underlying pathological mechanisms.^2^ However, HF cases can be broadly classified into two main types of similar incidence: HF with reduced ejection fraction (HFrEF, EF < 40%) and HF with preserved ejection fraction (HFpEF, EF > 50%).^2^ The lack of proper experimental models has greatly limited our understanding of HFpEF, although recent advances hold promise.^3,4^ On the other hand, multiple disease models are available to study the complexity and multifactorial origins of HFrEF.^3^

This article focuses on ischemic HF induced by MI due to its significance and ease of modeling. Myocardial infarction, primarily driven by coronary disease, impairs blood flow in the myocardium and results in cardiomyocyte cell loss. While reperfusion strategies have improved patient outcomes after MI, preventing future HF development caused by damaged heart tissue is challenging, particularly in the aging population. Post-MI, hearts experience electrical, structural, and metabolic remodeling, often leading to HFrEF.

Historically, researchers have leaned on animal models to study ischemic HF, primarily due to the straightforward approach of emulating MI by coronary artery ligation or occlusion. However, the confounding factors associated with surgical procedures aiming to replicate a progressive chronic human disease often modified by multiple risk factors and comorbidities, coupled with differences between animal and human physiology, hinder clinical translation. Multiple key points illustrate this challenge; for brevity, we present three examples. First, surgical trauma by thoracotomy in open-chest models of coronary artery ligation exacerbates inflammation within and outside the myocardium, limiting our understanding of immune cell regulation during MI.^5,6^ Secondly, anesthetics used in surgical procedures have been shown to promote ischemic pre-conditioning, which is known to reduce infarct size.^7,8^

Lastly, there are differences between animal and human HF that limit clinical translation. We can observe these limitations in the clinical trials for gene therapy replacement of sarcoplasmic reticulum calcium ATPase 2a (SERCA2a). Robust preclinical studies proposed SERCA2a downregulation as a hallmark of HF.^9,10^ Based on this premise, multiple clinical trials evaluated whether SERCA2a replacement therapy ameliorates HF. Yet despite promising preclinical studies, SERCA2a gene therapy failed to yield positive outcomes in patients.^11^^12^^13^ Recent evidence now clarifies this issue, indicating, with the largest proteomics data of cardiac tissues, that no differences in SERCA2a protein abundance are observed in patients with end-stage HF compared with control hearts.^14^ These examples underscore the complex nature of HF and clinical translation, highlighting the need for rigorous, multidiverse approaches to develop future effective therapies.

To break through existing barriers in translation, induced pluripotent stem cells (iPSC) offer a promising opportunity to study human cardiac biology, opening the path for personalized medicine. Human cells obtained from blood or skin biopsies can be reprogrammed to their pluripotent state using the four “Yamanaka factors,” a set of transcription factors first described in 2006,^15^ earning Dr. Yamanaka the Nobel prize in 2012. Thanks to their pluripotent state, iPSC can be differentiated into nearly all cardiovascular cell types. Differentiation of cardiomyocytes can be achieved by inducing mesoderm and cardiac lineage specification mimicking embryonic heart development. This can be achieved by dual modulation of the Wnt signaling pathway using growth factors or small molecules to obtain nearly pure populations of cardiomyocytes.^16^

**Figure 1:**
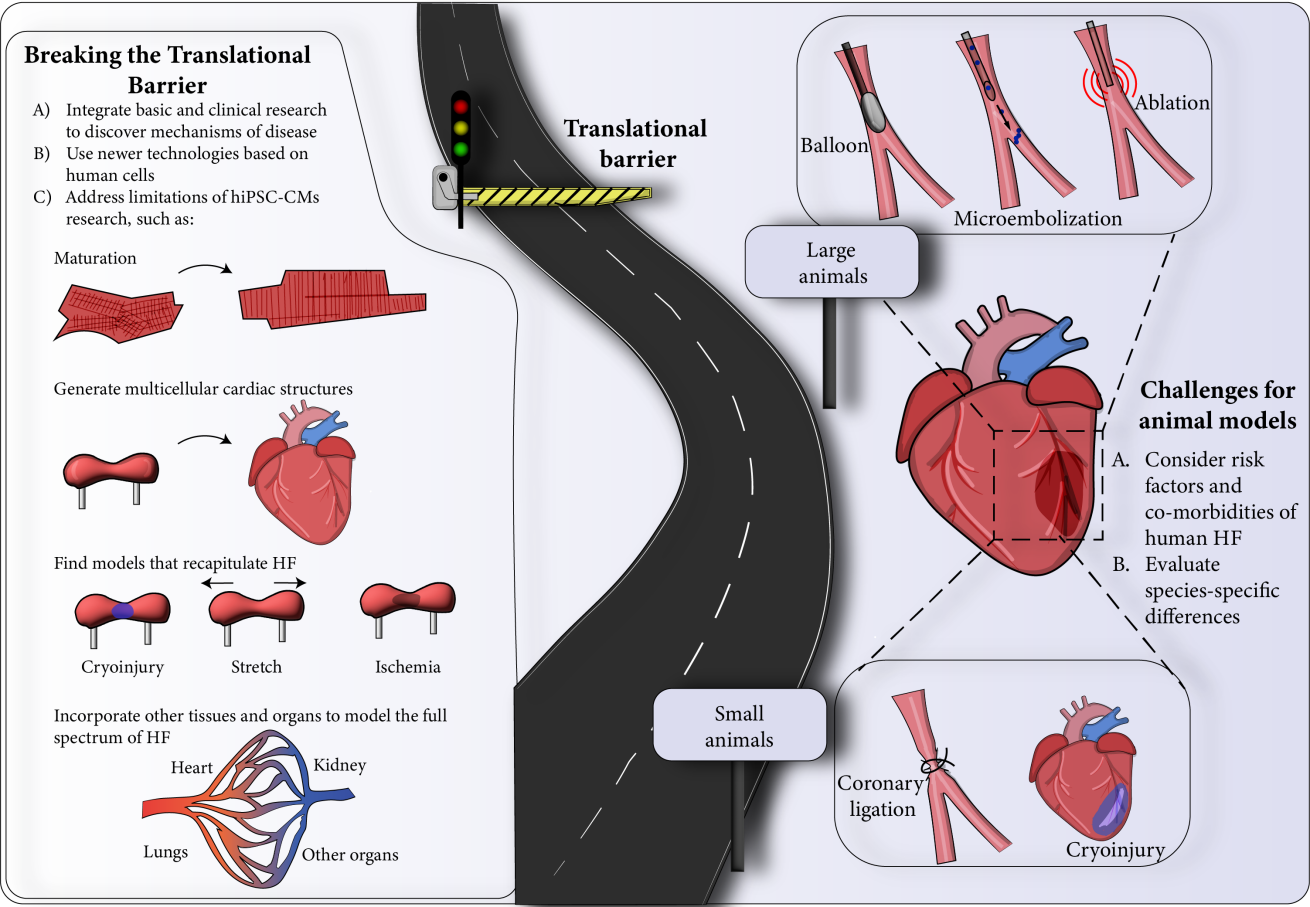
Strategies to advance research in heart failure. hiPSC-CMs: cardiomyocytes derived from human-induced pluripotent stem cells; HF: heart failure

In the next sections, we will briefly highlight the potential and limitations of animal models for HF research. Our focus then will shift to using hiPSC-CMs as disease modeling tools, discussing their strengths for precision medicine and limitations such as their immature state. Further in, we will focus on the utility of replicating the cardiac microenvironment using tridimensional hiPSC-CMs cultures to bridge the translational gap between animal models and humans while addressing the limitations of current engineered heart tissue (EHT) models. Finally, the discussion will converge on the potential of a comprehensive approach leveraging the strengths of small and large animal models and hiPSC-CMs to provide mechanistic rather than correlative evidence to accelerate the development of safe and effective HF therapies (Figure 1).

## Animal Models in HF Research

Multiple animal models have been developed to understand HF pathology and evaluate potential treatments. Our discussion primarily focuses on in vivo models of ischemic HF caused by MI. These models can be categorized into small and large animal models. Readers are referred to comprehensive reviews for a more exhaustive exploration of the literature regarding HFrEF and HFpEF animal models.^17,18^

### Small Animal Models

While various small animal models offer insight into HF, mice stand out as the most used model in the field. For readers seeking a more in-depth discussion of small animal models of heart failure, refer to Riehle et al.^19^ Mouse models have several advantages: They share genetic homology with humans, are easy to handle, have a short lifespan, and have low maintenance costs. Additionally, there are many genetic tools available that facilitate mechanistic research in mouse models, including deletion, overexpression, and gene-editing strategies that are useful for studying the molecular mechanism and genetics of HF.

MI can be achieved in mouse models by surgical ligation of the left coronary artery. Multiple variations of this procedure exist, including open-chest approaches with endotracheal intubation, minimally invasive non-intubating procedures, or closed-chest procedures aiming to reduce inflammation at the time of MI.^6,19,20^ Despite the fact this procedure recapitulates myocardium death, it lacks the human causes of MI like atherosclerosis and hypertension. Debate is ongoing about whether permanent or temporary occlusion better represents human pathology. Nonreperfused MI models enhance dilated remodeling and exacerbate dysfunction. From a clinical perspective, transient occlusion followed by reperfusion represents the most common clinical scenario in patients. Nowadays, only a small fraction of MI patients is not reperfused. For further discussion on this issue, including differences in scar size and cardiac dysfunction, readers are referred to Lindsey et al.^20^

Overall, mouse models are a reliable resource for discovering the molecular pathways involved in MI and HF. However, these models have various limitations, such as different cardiac anatomy and physiology compared to humans, highly inbred colonies with different fibrotic responses among species, and the fact that most studies are carried out in young, healthy mice.^19,21^ In addition, surgical interventions are difficult to standardize to obtain reproducible outcomes.

### Large Animal Models

Large animal models have emerged as invaluable tools to study the pathophysiology of MI and its progression to HF before clinical trials. For a state-of-the-art review of porcine, ovine, and canine models of HF, readers are referred to Silva et al.^18^

Because of their anatomical similarities to humans, catheter-based interventions permit minimally invasive and transient access to the coronary circulation to replicate human-like MI. Balloon-mediated angioplasty, cryo, electrical, or thermal ablation strategies, and coronary microembolization (injecting microspheres directly into the coronary circulation) allow localized ischemic events.^22^^23^^24^^25^ Open-chest models for MI using sutures are also available.^26^

There are several downsides to using large animals in research. First, their maintenance expenses are higher due to their longer lifespan. Second, ethical and cultural concerns have led to a decrease in their use. Third, genetically modifying these animals is complex, which limits the ability to study molecular mechanisms. Additionally, accurately modeling MI in large animals is challenging because they do not share the same risk factors and comorbidities as humans. Indeed, experiments are typically conducted on young and healthy animals, which may not accurately reflect the human condition. Lastly, differences in collateral coronary circulation and cardiac hemodynamics can impact study results. Therefore, while large animal models offer greater similarity to human MI, it is important to exercise caution when comparing findings from these models to human disease.

## Cellular Models to Study HF

Cellular models are useful for studying molecular pathways that confer cardioprotection after ischemia and reperfusion injury during MI. While current immortalized cardiomyocyte cell lines provide some insights, they do not fully emulate cardiomyocyte biology, leading to potential misinterpretations unless complemented with other cardiac disease models. HL-1 cell line, derived from an AT-1 mouse atrial cardiomyocyte tumor, has a gene expression pattern resembling atrial cells.^27^ H9C2 cell line, derived from embryonic rat tissue, exhibit a complex phenotype between cardiac and skeletal muscle.^28,29^ AC16 cell line, derived from the fusion of adult human ventricular cardiomyocytes with immortalized fibroblasts, expresses several cardiac contractile proteins but cannot assemble myofibrils.^30^ A recent comparative study showed that cardiac markers, transcriptomic profiles, ischemia and reperfusion injury, and hypertrophy response of cell lines do not resemble primary cardiomyocyte culture or adult cardiac tissue.^31^

Given the abovementioned limitations, primary cultures emerge as the next step toward studying cardiomyocyte biology. Frequently used neonatal rat ventricular cardiomyocytes (NRVM) are widely used to simulate in vitro ischemia and reperfusion injury. Although the technique is well-standardized and well-accepted in the field,^32^^33^^34^ NRVM are still immature. Consequently, to better understand the consequences of ischemia and reperfusion, researchers have turned to adult ventricular cardiomyocytes. Unfortunately, these experiments are limited by the extensive remodeling after isolation and the lack of tissue context. While it would have been ideal to perform more experiments in adult human cardiomyocytes, their limited availability and survival in culture have prevented this from happening. Cardiac slices obtained from transplanted hearts represent the closest model to study human cardiac function with improved survival in culture.^35^

Translating findings from animal models to humans has been challenging, which has increased the need for a new approach. This led to the development of hiPSC-CMs. Although these cells are human, they are immature. However, their human origin and the limitations of the abovementioned models make them a promising avenue for cardiac research. With their unlimited potential for maturation, hiPSC-CMs may revolutionize our understanding of HF. In the following sections, we explore the methods used to differentiate hiPSC-CMs, assess their potential in deciphering survival strategies after ischemia and reperfusion injury, and discuss how engineered heart tissues could be used to model heart failure.

## hiPSC-CMs as a Disease Modeling Tool

hiPSC-CMs have emerged as an efficient testbed for cardiotoxicity screening, drug discovery, disease modeling, and precision medicine development and validation. The first protocols for differentiated hiPSC had limited efficiency and reproducibility problems.^36,37^ Refined protocols are based on temporal modulation of the canonical Wnt signaling pathway with efficiencies over 95% in different hiPSC lines.^16,38^ Moreover, modern protocols allow massive expansion of hiPSC-CMs and biobanking of cardiomyocyte populations for later analysis and precision medicine.^39,40^ These methods provide researchers with an abundant supply of high-quality hiPSC-CMs to overcome past limitations, where only subpar cell lines were available to study cardiomyocyte biology. That said, hiPSC-CMs differ significantly from adult cardiomyocytes in their maturity. The challenge persists in refining these models to recapitulate the full spectrum of HF. In the subsequent sections, we explore the modeling of HF in hiPSC-CMs, their limitations, and how advanced methods could promote further maturation—aiming to realize the potential of “clinical trials” on a dish for HF.

## Modeling Heart Failure Triggers in hiPSC-CMs

Modeling human HF represents a major obstacle to discovering new therapies. Decoding and recapitulating molecular triggers of HF progression is a key step. One of the most common causes of HF is coronary artery disease, which narrows the functional diameter of oxygenated vessels, limiting oxygen supply and leading to ischemia and myocardial death.^41^ Although not covered in this article, hypertension can cause increased mechanical stress on the walls of the myocardium, leading to cardiac hypertrophy and, ultimately, HF.^42^ As the heart tries to maintain cardiac output after MI or chronic hypertension, its compensatory mechanisms become pathologic, leading to HF.^43^ To identify early signals promoting cardiac dysfunction, ischemic events and mechanical stress can be replicated in vitro.

Multiple mechanisms of cardiovascular disease can be recapitulated in hiPSC-CMs. Metabolically mature hiPSC-CMs are susceptible to hypoxia, inhibiting mitochondrial respiration and increasing cell death.^44^ Furthermore, mature hiPSC-CMs cells are vulnerable to hypoxia-reoxygenation damage, which mimics ischemia and reperfusion injury. As in the human heart, decreased pH during ischemia increases cell death even further, highlighting the efficacy of hiPSC-CMs as a novel in vitro model for developing a screening platform for new HF therapies.^45^

Researchers compared the gene expression profiles of hiPSC-CMs with NRVMs in culture under cyclic mechanical stretch and identified enrichment of biological processes and pathways related to cardiac hypertrophy.^46^ Moreover, mechanical stretch of human embryonic stem cell-derived cardiomyocytes increased cell size, expression of fetal genes, increased stiffness, and decreased contractility, all hallmarks of hypertrophic response.^47^ In addition to mechanical stretch, neuroendocrine molecules, upregulated in HF, are often used to simulate hypertrophic responses in NRVM cultures. Endothelin1 promotes a hypertrophic response in hiPSC-CMs characterized by increases in cell size and the expression of atrial and B-type natriuretic peptide.^48^ Moreover, hiPSC-CMs treated with norepinephrine and angiotensin II overexpressed genes associated with cardiac hypertrophy, similar to those found in failing human hearts.^49^ Altogether, hiPSC-CMs can help us unveil a deeper and more relevant understanding of human HF.

## Limitations of hiPSC-CMs Maturity

Although hiPSC-CMs offer a potential route for personalized medicine, they also pose various challenges. These cells do not have transverse tubules (T-tubules), invaginations of the plasma membrane that connect with the sarcoplasmic reticulum at the dyads to facilitate excitation-contraction coupling. Their sarcomeres are disorganized, and their resting membrane potential is more depolarized, leading to spontaneous activity. Metabolically, they primarily rely on glucose instead of lipids as the main source of fuel.^50^ For a comprehensive discussion of this topic, readers are referred to Karbassi et al. and Thomas et al.^50,51^ Much research, however, is being focused on improving hiPSC-CMs maturation to improve their utility and translation.

Based on the above, improved maturity in hiPSC-CMs opens the door to modeling and studying human HF response. For instance, using different plating methods increases maturity and contraction,^52,53^ which can help to study single-cell mechanisms. Thyroid and glucocorticoid hormones are essential in heart development. These hormones improve contractility force and calcium handling, increase higher maximal mitochondrial respiration, and promote the development of T-tubules in hiPSC-CMs.^54,55^ Also, combining these hormones with other components, such as palmitate, increases fatty acid oxidation metabolism and mitochondrial mass.^56^ Moreover, a simple addition of three fatty acids—palmitic, oleic, and linoleic acid—improves the maturity of hiPSC-CMs, increasing contraction force and mitochondria respiratory capacity.^57^ In summary, there are various methods for promoting maturation in hiPSC-CMs consisting of mechanical, electrical, hormonal, and metabolic cues. Considering these challenges and strategies, the search for methods to fully maturate hiPSC-CMs into functional adult cardiomyocytes is ongoing.

HF affects the entire heart, not just cardiomyocytes; thus, EHTs have been developed to include additional cell types. The use of hiPSC-CMs and human cardiac fibroblasts to engineer 3-dimensional (3D) tissues has been an advantage, improving hiPSC-CMs maturity and creating a multicellular model. Ronaldson-Bouchard et al. assembled hiPSC-CMs with supporting fibroblasts and, following electrical stimulation, demonstrated more organized cellular ultrastructure, increased mitochondrial density and fatty acid oxidation, the presence of T-tubules, and more physiological calcium handling.^58^ Another group created cardiac microtissues using hiPSC-CM, human cardiac fibroblast, and human endothelial cells and achieved greater maturity with thyroid hormone, dexamethasone, and insulin-like growth factor-1 treatment. These cardiac tissues resembled adult ventricular cardiomyocyte electrophysiological and calcium properties with improved ultrastructure and contractile function.^59^

## Are Three-dimensional Models the Solution to Bridging the Translational Gap?

Engineered heart tissues have emerged as a powerful testing platform, filling some of the voids left by animal models and hiPSC-CMs monolayers. These 3D structures, composed of cardiomyocytes and other cardiac cells, aim to recapitulate the cardiac microenvironment, paving the way for more accurate disease modeling. EHTs take several forms, some of which specialize in the study of contractile force and dynamics, and some of which excel at integrating varied cell types to observe cell-cell interactions. Pillared EHTs consist of a thin strip of cardiomyocytes, usually with fibroblasts, mounted between two silicone pillars. The passive resistance provided by the pillars can be adjusted to recapitulate the hemodynamic changes and myocardial dysfunction observed in HF.^60^^61^^62^ On the other hand, cardiac spheroids, self-assembling cell assemblies, vary greatly in diameter with greater sizes creating an ever-larger necrotic core where oxygen and nutrients struggle to diffuse in and waste out, causing the interior cells to die.^63,64^ Cell waste and debris diffuse out of the necrotic core, providing a persistent ischemic state. While undesirable to model other diseases, and methods to reduce the necrotic core do exist, ischemia is a critical part of HF development and may be beneficial in this case.^65^

Early EHTs consisted of only cardiomyocytes, but more recently, the inclusion of other cell types has been used to model cell-cell interactions in varied physiological and pathophysiological states.^66^ The addition of cardiac fibroblasts and the extracellular matrix they deposit increases cardiomyocyte maturation survival and provides significant cell-cell interaction between the two types.^63,67^ Vascular pathology is a critical component and therapeutic target of HF, and the addition of endothelial cells has allowed the examination of endothelial dysfunction.^68^ Key to the study of HF neuroendocrine genesis, neural progenitor cells improve contractile dynamics and form a connected network throughout the spheroid.^69^ Overshadowed by their far more numerous cardiomyocytes, smooth muscle cells are also present in the heart and improve cardiomyocyte maturation, contractile dynamics, and bioenergetic production.^64^ For cardiac spheroids specifically, several groups have layered different cell types in addition to mixing, allowing more granular control over which cell types interface with each other.^70^ Importantly, cardiomyocytes are not crowded out by neighbor cells and typically occupy more spheroid volume as they mature with cocultured EHTs, often outlasting pure cardiomyocyte EHTs.^71^

EHTs allow the assessment of several triggers of heart injury. For instance, EHT opens the possibility of studying cardiac regeneration as a treatment for MI. EHT can recover functionally after cryoinjury, allowing for assessment of cardiac regeneration and remodeling post-injury.^72^ Interestingly, a 3D-engineered tissue formed by endothelial, epicardium, and cardiac cells adopts a chamber-like structure, which, when submitted to cryoinjury, produces a more physiological response and helps to understand the role of extracellular matrix in HF pathology.^73^ Also, EHT modelling of ischemia and reperfusion injury helps to evaluate the effects of cardioprotective factors for improving translation into the clinic.^74,75^ Recently, Richards et al. showed that cardiac organoids under oxygen-diffusion gradient and norepinephrine stimulation mimics the gradient of an infarct, border, and remote zones, observed in heart tissue after MI.^75^ Moreover, new methods are being developed to investigate additional factors contributing to HF, including the use of senescent hiPSC-CMs populations for EHT formation to mimic aging.^76^

Altogether, the advantages of bioengineered tissues create more sophisticated human cellular models to study MI and HF, cardioprotective factors, and molecular pathways with therapeutic purposes.

## Conclusion

HF is a complex disease with systemic effects. The initial compensatory mechanisms for the heart after injury gradually give rise to their own pathophysiological state, exacerbating cell and organ stress across the body. Current therapies fail to address all components of HF and as global burden and incidence increase, there is an urgent need for safe, effective treatment options. A deeper molecular understanding of the underlying mechanistic processes of HF will better equip researchers to develop and validate such therapies, accelerating translation to clinical practice.

Currently, there is no perfect human heart experimental model, and there may never be. However, many of the strengths and weaknesses of animal models and hiPSC-CMs complement each other and together can provide deeper mechanistic insight than either could alone. Tridimensional hiPSC-CM–based culture systems have proven useful in isolating processes for targeted examination. Integration of other cell types impacted by HF, including fibroblasts, endothelial cells, and smooth muscle cells, has revealed cell-cell interactions that may be impossible to discern in humans or animal models. Further improvements in hiPSC-CMs production and maturation techniques will enable more faithful recapitulations of cardiovascular diseases. The strategic integration of animal models with hiPSC-CMs helps to reinforce and demonstrate the importance of experimental findings, hopefully improving clinical translation of novel therapeutic targets of heart failure.

## Key Points

- Small and large animal models used in the study of HF have limitations in fully modeling human disease, leading to challenges in clinical translation.
- Cardiomyocytes derived from human induced pluripotent stem cells (hiPSC-CMs) and engineered heart tissues (EHTs) offer innovative solutions to study human genetic and molecular mechanisms driving cardiomyocyte dysfunction and death in heart failure (HF).
- A key challenge is refining hiPSC-CMs and EHT technologies to better replicate the complete physiological environment of the human heart and address their current maturation limitations.
- A collaborative research approach that integrates mechanistic insights from both animal and hiPSC-CMs models and rigorous clinical research will enhance our understanding of human HF and aid in developing more effective therapies.
